# Increased Gray Matter Diffusion Anisotropy in Patients with Persistent Post-Concussive Symptoms following Mild Traumatic Brain Injury

**DOI:** 10.1371/journal.pone.0066205

**Published:** 2013-06-11

**Authors:** Sylvain Bouix, Ofer Pasternak, Yogesh Rathi, Paula E. Pelavin, Ross Zafonte, Martha E. Shenton

**Affiliations:** 1 Department of Psychiatry, Brigham and Women’s Hospital, Harvard Medical School, Boston, Massachusetts, United States of America; 2 Department of Radiology, Brigham and Women’s Hospital, Harvard Medical School, Boston, Massachusetts, United States of America; 3 Department of Physical Medicine and Rehabilitation, Spaulding Rehabilitation Hospital, Harvard Medical School, Boston, Massachusetts, United States of America; 4 Department of Physical Medicine and Rehabilitation, Massachusetts General Hospital, Harvard Medical School, Boston, Massachusetts, United States of America; 5 Department of Psychiatry, Veterans Affairs Boston Healthcare System, Brockton Division, Brockton, Massachusetts, United States of America; West China Hospital of Sichuan University, China

## Abstract

A significant percentage of individuals diagnosed with mild traumatic brain injury (mTBI) experience persistent post-concussive symptoms (PPCS). Little is known about the pathology of these symptoms and there is often no radiological evidence based on conventional clinical imaging. We aimed to utilize methods to evaluate microstructural tissue changes and to determine whether or not a link with PPCS was present. A novel analysis method was developed to identify abnormalities in high-resolution diffusion tensor imaging (DTI) when the location of brain injury is heterogeneous across subjects. A normative atlas with 145 brain regions of interest (ROI) was built from 47 normal controls. Comparing each subject’s diffusion measures to the atlas generated subject-specific profiles of injury. Abnormal ROIs were defined by absolute z-score values above a given threshold. The method was applied to 11 PPCS patients following mTBI and 11 matched controls. Z-score information for each individual was summarized with two location-independent measures: “load” (number of abnormal regions) and “severity” (largest absolute z-score). Group differences were then computed using Wilcoxon rank sum tests. Results showed statistically significantly higher load (p = 0.018) and severity (p = 0.006) for fractional anisotropy (FA) in patients compared with controls. Subject-specific profiles of injury evinced abnormally high FA regions in gray matter (30 occurrences over 11 patients), and abnormally low FA in white matter (3 occurrences over 11 subjects). Subject-specific profiles provide important information regarding the pathology associated with PPCS. Increased gray matter (GM) anisotropy is a novel *in-vivo* finding, which is consistent with an animal model of brain trauma that associates increased FA in GM with pathologies such as gliosis. In addition, the individualized analysis shows promise for enhancing the clinical care of PPCS patients as it could play a role in the diagnosis of brain injury not revealed using conventional imaging.

## Introduction

More than 1.7 million people each year in the United States experience a traumatic brain injury (TBI), with 75 to 85% of these categorized as mild (mTBI). It is estimated that 5–15% of mTBI patients suffer from persistent post-concussive symptoms (PPCS) that do not resolve, and can lead to long-term disabilities [1]–[4]. In the majority of these chronic cases, there is no radiological evidence for injury in CT or anatomical MRI, and the pathology related to prolonged symptoms is not known, or it is assumed to be of psychogenic origin [5]. However, it is likely that the imaging methods used are not sufficiently sensitive to the type, size, or pattern of possible chronic pathologies [6]–[8].

A promising approach to increase the sensitivity to subtle pathologies is diffusion tensor MRI (DTI), which can measure microstructural tissue changes [9]. However, detecting DTI abnormalities in PPCS subjects is challenging because the impact of the injury is very heterogeneous, and is unlikely to affect the brains of different subjects in exactly the same way [10], [11]. Unfortunately, standard population analysis tools assume a common spatial pattern of pathologies over the *entire patient group*. As such, they have limited sensitivity. This is also reflected in recent reviews of DTI findings in mTBI that highlight the variability of findings in mTBI and PPCS [6]–[8], with the most consistent finding being decreased Fractional Anisotropy (FA) in white matter (WM) related to chronic diffuse axonal injury (DAI) in PPCS.

DTI is well-known for its ability to reveal abnormalities in WM fiber structure and to provide models of brain structural connectivity [12]. As a consequence, most DTI analysis tools are specifically designed to study WM and do not investigate GM regions [13], [14]. Nevertheless, a few DTI studies report increased diffusivities in GM, which correspond to macrostructural GM alterations in PPCS demonstrated in structural MRI reports [15], [16]. Although a recent animal model observed increased FA in GM two months following TBI, which was linked to gliosis, there are no previous reports of increased FA in GM linked to prolonged symptoms in humans [17].

In this study, we compared diffusion MRI in a cohort of PPCS patients following mTBI and matched controls. We proposed a novel atlas-based individualized analysis in order to overcome the heterogeneity challenge and to detect abnormalities that are *unique to each patient*. We sought to extend the DTI analysis into GM. We further sought to combine individual maps for group comparisons, and to investigate potential common patterns of abnormalities in WM and GM.

## Methods

### Ethics Statement

The research was conducted according to the principles expressed in the Declaration of Helsinki, and approved by Brigham and Women's Hospital and Spaulding Rehabilitation Hospital Institutional Review Boards (protocol number: 2008P001834). All participants gave written informed consent.

### Study Design and Participants

Eleven patients with PPCS following mTBI were recruited for this study. Injury was secondary to motor vehicle accident, blast exposure, sports-related injury, and assault (Table 1). All subjects were initially diagnosed with mTBI with a Glasgow Coma Scale of 13 to 15. The average duration-since-injury was 62.08±46.35 months. All subjects demonstrated persistent symptoms such as headaches, emotional dysregulation, or cognitive and memory impairments at the time of the evaluation. Patients did not exhibit radiological findings in CT or anatomical MRI at recruitment.

**Table 1:** Description of Individual PPCS subjects.

Case Number	Age	Gender	Source of Injury	Duration Since Injury (months)	Symptoms self-report	FA Abnormalities Summary
TB01	45	F	MVA*	17.0	Cognitive impairment, emotional dysregulation, depression.	GM: **left amygdala (3.54^ t^)**
TB02	38	M	MVA	106.6	Mild memory impairment, mild executive function impairment, emotional dysregulation, depression.	GM: **left superior temporal gyrus (**−**3.48^t^)**
TB03	44	F	MVA	121.3	Dizziness, exhaustion, hypersomnia, depression and anxiety, periodic limb movements.	WM: **right medial orbito frontal gyrus (**−**2.88^t^)**
TB04	30	M	Sports injury	2.6	Diplopia, fatigues easily, executive function impairment.	GM: left caudal anterior cingulate (3.73)WM: **left corpus callosum (**−**4.47)**, right corpus callosum (−3.82)
TB05	42	M	MVA	138.0	Cognitive impairment, memory and executive function.	WM: **right corpus callosum (**−**4.15)**
TB06	28	M	Assault	27.0	Anxiety, depression, insomnia, irritability, ADHD, intrusive thoughts, memory deficits, overeating.	GM: **left entorhinal (6.58)**, left fusiform (4.37), left inferior temporal (4.08), left lateral occipital (3.83)
TB07	24	M	Blast Exposure	70.3	Anxiety, panic attacks, hypervigilance, overeating, difficulty concentrating.	GM: **left lateral orbito frontal (3.39^t^)**
TB08	25	M	Blast Exposure	83.3	Depression, difficulty w/rapidly presented information, memory impairment.	GM: **right hippocampus (5.01)**, left thalamus (3.58), right thalamus (3.81), right putamen (4.82), right amygdala (4.07), left inferior parietal (3.77), left lateraloccipital (4.28), left lingual (3.66), left parahippocampal (3.80), left precuneus (4.21), right lateraloccipital (3.97), right lingual (4.56), right parahippocampal (3.93), right pericalcarine (4.30), right precuneus (4.30)
TB09	29	M	Blast Exposure	51.4	Irritability, nightmares, panic attacks, depression/anxiety, difficulty concentrating, cognitive and memory impairments.	GM: **left entorhinal (4.81)**
TB10	24	M	Blast Exposure	55.9	Headaches, memory impairment, problems concentrating, anxiety, irritability, nightmares.	GM: **left paracentral (4.36)**
TB11	39	M	Sports Injury	9.5	Facial pain, difficulty concentrating, emotional dysregulation, memory and executive function impaired.	GM: **right putamen (4.62),** left amygdala (3.81), left lateral orbito frontal (4.22), left parahippocampal (3.60), left pericalcarine (3.74), left superiortemporal (4.22), right supramarginal (3.67)

* MVA = Motor Vehicle Accident; In the FA abnormality summary column z-score are given in parenthesis, the highest severity score is in a bold font, ^t^ - is affixed to z-scores who did not reach significance.

The patients were individually compared with a normative atlas that was built from an imaging database of 47 normal controls (NC) who underwent the same MRI protocol. Of these 47, eleven were selected to closely match the PPCS patients on age and gender (Table 2).

**Table 2:** Demographic characteristic of study subjects.

	Atlas Normals (n = 47)	Matched Normal Controls (n = 11)	PPCS (n = 11)	p (test) between matched NC (n = 11) and PPCS (n = 11)
**Age in years, mean(SD) [range]**	32.3(11) [19–52]	32.1(8.5) [23–47]	33.3(8.4) [24–45]	0.98 (t-test)
**Gender (male/female)**	33/14	10/1	9/2	1 (Fischer exact)
**Handedness**	0.41(0.48)	0.43(0.79)	0.69(0.45)	0.55 (t-test)
**Years of education**	14.6(2.4)	15.4(1.9)	14.5(2.6)	0.56 (t-test)
**Parental Socioeconomic status**	2.21(0.99)	2.44(1.01)	2.76(1.09)	0.48 (t-test)

The exclusion criteria for both PPCS and normal subjects were: sensory-motor impairments, apparent psychiatric disorder, seizure disorder, prior neurosurgical procedures, medical illnesses that significantly impaired neurocognitive function, and contraindications for MRI. In addition, control subjects were screened to exclude individuals who had a brain injury, prior history of concussion, or any clinical/cognitive symptoms at the time of the evaluation.

### Procedures

All PPCS participants completed a standardized clinical neuropsychological assessment and an MRI session on the same day. The neuropsychological tests tapped into areas of dysfunction in PPCS. This battery included the following tests: the California Verbal Learning Test II [18]; the Processing Speed Index; Digit Span [19]; the Trail Making Test part A and Trail Making Test part B [20]; the Controlled Oral Word Association Test [21]; and the Stroop Test part 1 and Stroop Test part 2 [22].

DTI data was acquired on a 3 Tesla GE Echospeed system (General Electric Medical Systems, Milwaukee, WI). Scans were acquired with an echo planar imaging (EPI) DTI sequence, and a double echo option to reduce eddy-current related distortions. To reduce the impact of EPI spatial distortion, an 8 Channel coil and ASSETT (Array Spatial Sensitivity Encoding techniques, GE) with a SENSE-factor (speed-up) of 2 was used. 85 axial slices parallel to the AC-PC line covering the whole brain were acquired in 51 diffusion directions with b = 900 s/mm^2^ that is well suited for ROI-based DTI analyses. We chose not to increase the b-value further in order to maintain higher SNR [23]. Eight baseline scans with b = 0 were also acquired. Scan parameters were as follows: Repetition time (TR) of 17000 ms, echo time (TE) of 78 ms, field of view (FOV) of 24 cm, with a 144×144 matrix, and 1.7 mm slice thickness, producing isotropic voxels. Total scanning time for the DTI sequence was 17 minutes. In addition to the DTI scan, a structural MRI acquisition protocol was used, which included two MRI pulse sequences. The first resulted in contiguous spoiled gradient-recalled acquisition (fastSPGR) with the following parameters; TR of 7.4 ms, TE of 3 ms, inversion time (TI) of 600 ms, 10 degree flip angle, FOV of 25.6 cm, and a 256×256 matrix, with voxel dimensions of 1 mm×1 mm×1 mm. The second- XETA (eXtended Echo Train Acquisition) produced T2-weighted images (TR = 2500 ms, TE = 80 ms, FOV = 25.6 cm). Voxel dimensions were also 1 mm×1 mm×1 mm. This latter sequence was used as the additional channel of information for brain segmentation.

Motion and artifacts in the diffusion data were corrected using affine registration of all gradient volumes with the first b = 0 volume (FLIRT; FMRIB Software Library, Oxford, UK), and gradients directions were compensated for rotations [24]. The transformation matrices were used to estimate a relative-motion parameter [25]. No differences were found in this parameter between controls and PPCS (t-test, p = 0.51).

Diffusion tensors were estimated using in-house software based on a linear least square fit [9], with an added procedure to correct tensors with negative eigenvalues. The fit was performed iteratively. At each iteration, the algorithm identified voxels that had tensors with one or more negative eigenvalues. The diffusion-weighted images were then smoothed at that voxel location using the neighboring voxels, which did not have negative eigenvalues. The following DTI indices maps were then calculated for each subject: Fractional Anisotropy (FA), Mean Diffusivity (MD), Axial Diffusivity (AD) and Radial Diffusivity (RD). Each of these measures offers different insights into potential pathologies affecting the cellular environment in which water diffuses. In WM, these measures have been associated with pathologies such as edema, axonal damage or demyelination [26]. However, little is known about their association with GM microstructural properties.

Each T1 image was parcellated using FreeSurfer [27], resulting in 176 GM, WM, and cerebrospinal fluid (CSF) sections. CSF sections and sections smaller than 300 mm^3^ were excluded from the analysis. The remaining 145 sections –83 in GM and 62 in WM – were mapped onto the diffusion space by rigidly registering the T1 image with the T2 image, non-linearly registering the T2 image with a b = 0 image, and using the composite transformation to co-register the FreeSurfer parcellation.

In contrast to population-based analyses, our approach detects abnormalities in a *single patient* by building a normative atlas and then testing *each individual* PPCS patient against the atlas. The normative atlas captures the distribution of DTI indices (FA, MD, AD, RD) within the entire sample of NC for selected ROIs. As age has been documented to have an effect on DTI measures [28], we corrected the DTI indices for age before building the statistical atlas. The correction was performed with a Generalized Linear Model fitting procedure to estimate the linear regression between age and DTI indices in normal subjects only (n = 47) [29], We found 47/145 regions showed statistically significant correlations (p<0.05). The extracted regression coefficients were used to correct the DTI indices for age in all subjects (including mTBI participants). The normative atlas was represented by the sample mean and standard deviation over the NC population, calculated for each age-corrected feature in each ROI. Note that the age correction coefficients are computed only from the normal population and applied to both PPCS and control subjects. Our rationale for this procedure is that we treat a new subject as if it were normal and then test whether its derived measures are outside the normal range. If the abnormality stems from different age regression coefficients for PPCS, we would rather detect them than correct for them.

A subject-specific profile was generated by calculating a z-score for each DTI index against the corresponding normal distribution as represented by the atlas. The cause of the abnormality could be due to an underlying age/time-dependent phenomenon.

Abnormal ROIs were defined as those that had absolute z-score values above a given threshold. The main statistical consideration in setting the threshold was the effect of multiple comparisons on finding false abnormal ROIs. We used Bonferroni correction to adjust the z-score threshold from |z|>1.96 to |z|>3.58 (p<0.05 corrected for 145 tests).

We note that a non-parametric approach might also be suitable to detect abnormal regions, however we found 165 out of 176 ROIs to have normal distribution of age corrected FA across the 47 normal control subjects (Lilliefors' composite goodness-of-fit test). As parametric tests are usually more powerful for normal distributions than non-parametric ones, and z-scores are well understood in clinical diagnosis and easy to interpret, we decided to use this parametric method for our analysis.

### Statistical Analysis

In all of the experiments we performed, the entire group of 47 controls was used to generate the atlas. In order to test the sensitivity and specificity of the atlas, we chose 11 controls group matched for age, gender, education and PSES to the PPCS cohort (**Table 2**). We then tested these 22 subjects against the complete atlas, and then compared the results of the test using group statistics. Of note, when testing a control subject against the atlas we used a leave one out approach, i.e., we compared that subject with an atlas composed of all 47 normal individuals minus the normal subject being tested. In essence, we were testing whether the patients are “more different” than their 11 well-matched controls when compared to a large set of normal individuals with a wide range of demographics.

In order to perform group comparisons not affected by heterogeneous injury locations, z-score information for each individual was made location-independent by summarizing it with two measures: “load” (number of abnormal ROIs) and “severity” (largest absolute z-score). To compare the location-independent summary measures between groups we performed a two-sample Wilcoxon rank sum tests. The need for a non-parametric test stems from the distribution of the “load” parameter, which is not normal, as it is strictly positive and expected to have mean/median values very close to zero in controls, and to have a wide and heterogeneous range of values in patients.

Subject-specific profiles for subjects can also be summarized by displaying them as scatter plots where the x-axis represents anatomical labels corresponding to the FreeSurfer sections and the y-axis is the raw z-score.

To identify whether or not abnormalities are common across the PPCS patients, we computed the frequency of abnormalities per ROI. This was done by counting the number of subjects that had a z-score higher than the threshold in a particular location and for a particular feature.

In addition to the atlas-based analysis, we performed conventional group comparison using two-sample Wilcoxon rank sum tests to identify group differences in age corrected DTI indices of each ROI between the PPCS and NC groups. The significance thresholds were adjusted for the multiple comparisons of ROIs using Bonferroni correction.

Non-parametric Spearman correlations were calculated to investigate correlations between FA location-independent summary measures (severity, load), the neuropsychological scores, and duration since injury. The significance threshold of the correlation was set to p<0.05. Due to the small number of subjects, this is an exploratory analysis and correlations were not corrected for multiple comparisons.

## Results

### Conventional Group Comparisons

Wilcoxon rank sum tests on each DTI index in each ROI between PPCS and matched NCs were not statistically significant (p>0.05; Bonferroni corrected) for any of the measures (FA/MD/AD/RD).

### Subject-specific Profile

**Figure 1:**
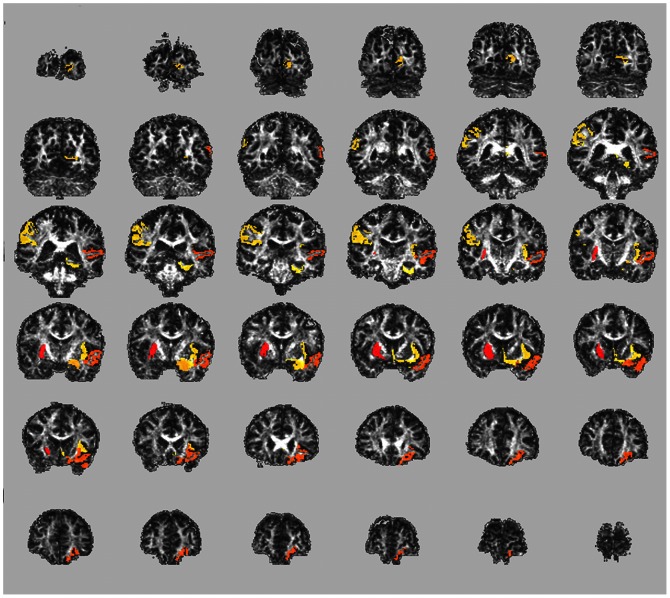
Subject-specific abnormality map. A subject-specific abnormality map of an individual PPCS subject (TB11) shows ROIs with abnormal FA values compared with the normative atlas. These abnormalities were not detected in clinical CT or MRI scans, nor were they detected using a conventional group comparison. Coronal slices spanning the entire brain are presented (Posterior–top left to Anterior–bottom right). Abnormalities are highlighted in color (yellow to red, for lower to higher z-scores) overlaid on top of an FA map of this subject. This subject had the following ROIs highlighted as abnormal: Right Putamen (z = 4.62), Left Lateral Orbitofrontal Cortex, (z = 4.22), Left Superior Temporal Gyrus (z = 4.22), Left Amygdala (z = 3.81), Left Pericalcarine Cortex (z = 3.74), Right Supramarginal Gyrus, FA (z = 3.67) and Left Parahippocampal gyrus (z = 3.60).

**Figure 1** shows an example of a subject-specific FA abnormality map of a single PPCS patient. Similar maps of all DTI measures were produced for all PPCS subjects and their matched NCs. These maps highlight areas of the brain for which this patient had an “out-of-normal-range” value and offer an intuitive way to assess individually the location of injury. In contrast to conventional group testing, when comparing the location-independent summary measures of load and severity between PPCS and NC groups for FA, the PPCS group had significantly higher load (Wilcoxon rank sum; p = 0.018), and significantly higher severity (Wilcoxon rank sum; p = 0.006). MD, RD and AD did not show significant differences between the two groups. Consequently, all subsequent analyses focus solely on FA.

**Figure 2:**
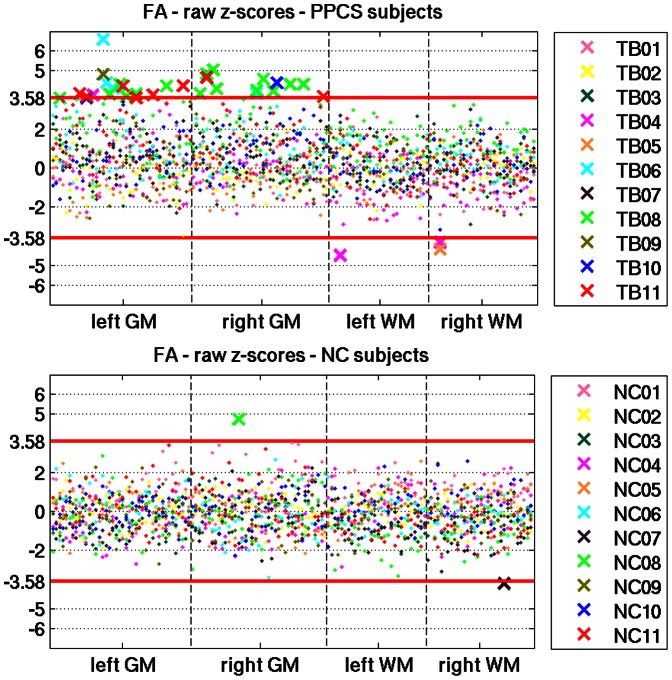
Raw z-scores for all subjects. Scatter plots of the raw z scores for FA for all PPCS patients (top) and their controls (bottom). An ‘x’ represents ROIs that are significantly different than the normative atlas (higher or lower than the Bonferroni corrected z-score threshold of ±3.58). Non-significant regions are represented as dots. When FA is abnormal in the GM of PPCS patients it is always higher than the normative atlas. When it is abnormal in the WM of PPCS patients it is always lower than the normal range. There were many more abnormalities detected in GM than in WM. There were only 2 abnormal regions across all of the NC population.

In **Figure 2**, we summarize all of the individual FA abnormality maps into a scatter plot, with the x-axis representing the FreeSurfer ROIs, and the y-axis representing the raw z-scores. Each subject is assigned a unique color, in order to visualize potential patterns that repeat across subjects. The abnormal regions are specified in **Table 1** **.** First, the PPCS population has more abnormal scores than the NC group (33 in PPCS, and 2 in NC). Furthermore, the PPCS population shows two distinct patterns of abnormal FA: When *FA is abnormal in GM it is always higher than the normative atlas*; when it is abnormal in WM it is always lower than the normal range. Finally, PPCS patients had more abnormalities in GM (30 high z-scores found in 26 out of 83 GM regions) than in WM (3 low z-scores found in 2 out of 62 WM regions).

Within GM, the PPCS population demonstrates *heterogeneity* in the location, magnitude and quantity of detected abnormalities. Among the 26 ROIs with abnormally increased FA, only four are present in more than one mTBI subject. These ROIs are: left accumbens, right putamen, mid anterior and left precuneus; each found abnormal in two different patients.

### AD and RD in ROI with Abnormal FA

To investigate further the source of FA abnormalities, we evaluated AD and RD in the ROIs with “out-of-normal-range” FA. In WM, across the 11 PPCS patients, there were three abnormal z-scores spanning two ROIs, all with FA lower than normal. The corresponding RD in these regions had high positive z-scores (+3.89, +3.07 and +2.25). The corresponding AD z-scores were within normal range (+1.26, +0.71 and −0.87).

**Figure 3:**
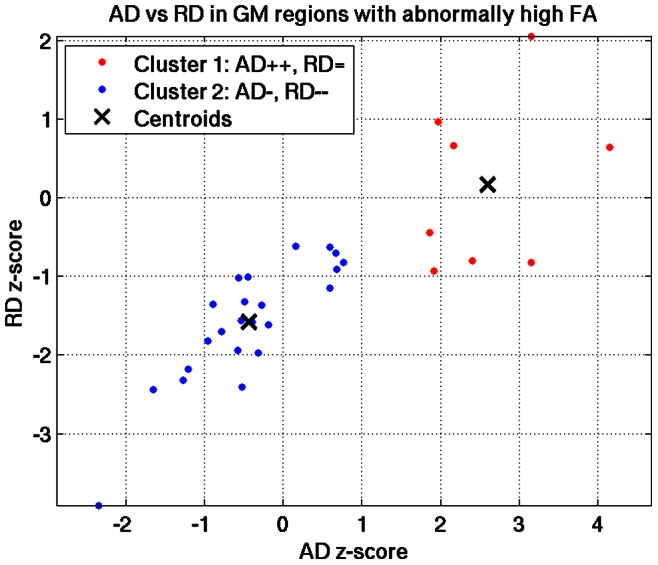
Axial diffusivity (AD) vs. Radial diffusivity (RD) in GM ROIs with abnormally high FA. There are two patterns that can explain the increased FA evinced in GM of PPCS patients. In Cluster 1 (red) AD is abnormally high and RD is normal, consistent with an animal model of gliosis. In Cluster 2 (blue) both AD and RD are abnormally low, a pattern that was not reported in previous GM studies and which remains to be explained.

In GM, across the 11 PPCS patients, there were 30 abnormal z-scores spanning 26 ROIs, all with FA higher than normal. When plotting the corresponding AD vs. RD values (**Figure 3**), we observed several behaviors and thus ran a k-means analysis to separate the data into clusters [30]. We tested separating the data using two to ten clusters and used the silhouette measure to select the optimum number of clusters, which was two (colored in red and blue in **Figure 3**) [31]. We further investigated whether or not the median AD and RD in each cluster was equal to zero, and found the following behavior: Cluster 1) significantly increased AD z-score (Wilcoxon signed rank; p = 0.008) and no change in RD (Wilcoxon signed rank; p = 0.84); and Cluster 2) negative AD z-score (Wilcoxon signed rank; p = 0.04) and negative RD z-score (Wilcoxon signed rank; p<0.0001). These results suggest that the observed elevated FA in GM may be caused by two different mechanisms.

### FA and PPCS Symptoms

We tested for correlations between all neuropsychological measures and both the severity and load measures. There was a significant negative correlation between the digit symbol test and both FA severity (Spearman correlation; rho = −0.642; p = 0.033) and load (Spearman correlation; rho = −0.617; p = 0.033). In addition, a trend level negative correlation was observed between duration-since-injury and FA severity (Spearman correlation; rho = −0.555; p = 0.077), which was significant when tested with a Pearson correlation (R = −0.645; p = 0.032). We did not find significant correlations between the FA measures and any of the other neuropsychological scores (**Table 3**), however the population sample was small.

**Table 3:** Correlations between neuropsychological test and FA severity for PPCS patients.

		FA Severity		FA Load	
Test	Subtest	Spearman’s rho	Significance (2-tailed)	Spearman’s rho	Significance (2-tailed)
**Duration since injury**		−*0.555*	*0.077*	−0.430	0.187
**California Verbal Learning Test II**	Trials 1–5 Total	−0.046	0.894	0.077	0.822
	Short Delay Free Recall	−.376	.254	−.415	.205
	Short Delay Cued Recall	−.467	.148	−.340	.306
	Long Delay Free Recall	−.398	.225	−.298	.373
	Long Delay Cued Recall	−.369	.265	−.237	.482
**Processing Speed**	Digit Symbol	−.**642** *	**.033**	−.**617** *	**.043**
	Symbol Search	−.061	.859	−.211	.534
	Processing Speed Index - Percentile	−.121	.723	−.240	.478
**Digit Span**	Digit Span	−.438	.178	−.422	.197
	Digit Span - forward	−.372	.261	−.347	.295
	Digit Span - backward	−.364	.271	−.368	.265
**Trail Making**	Trail Making Test A	.247	.464	.177	.604
	Trail Making Test B	.330	.322	.445	.171
**Controlled Oral Word Association Test**		.224	.507	−0.96	.778
**STROOP**	Probability of a score being obtained by a person with brain damage	−.*511*	.*131*	−.228	.526

* Scores with significance level p<0.05 are in a bold font.

## Discussion

Our findings demonstrate that PPCS following mTBI may have a physiogenic origin that could not be observed by a neuroradiologist using clinical MRI, but that can be detected by applying a novel subject-specific atlas using DTI data. Specifically, we found that the PPCS population had spatially heterogeneous FA abnormalities. Furthermore, these microstructural abnormalities were most prominently in GM.

Due to the heterogeneity of the injuries, abnormalities in the PPCS group were detected only when using the normative atlas, suggesting that the location of abnormal ROIs is *unique to each subject,* a result consistent with other recent reports [11], [32]. In addition, we believe our ROI based analysis benefits from stronger statistical power than voxel-based approaches such as TBSS, as we analyze about 150 regions as opposed to thousands of voxels. Furthermore, using bigger regions (ROIs) diminishes the effect of misregistration. Perhaps more importantly, due to the inherent variability of brain morphometry, voxel-based techniques most often restrict their analyses to core white matter structures, while our method investigates the entire brain, including both gray and white matter areas.

The location-independent summary measures (load and severity) derived from subject-specific maps, further revealed significant group differences when comparing PPCS patients with NCs. In contrast, differences could not be found using conventional group comparison tests in each ROI. These findings support the hypothesis that there is an underlying pathology that is detectable in the PPCS group, and that subject-specific methods are essential to detect such pathologies. We also found that of the measures tested, FA is the most sensitive for detecting abnormal brain regions in PPCS.

The most repeated pattern that emerged was an increase in FA in GM of PPCS subjects following mTBI. Moreover, there were less abnormally high FA values in GM for subjects who had higher scores in the digit symbol test. This strengthens the association between abnormally high FA and PPCS, since the digit symbol test is sensitive to neurological dysfunction [22].

In reviewing the literature, we could not find other PPCS or even TBI studies that report increased FA in GM, except for one recent animal study by Budde et al. [17], which offers an interesting hypothesis linking increased FA in GM two months following mTBI with a proliferation of glial fibrillary acidic proteins - a marker for gliosis. In that study, the increase in FA related to gliosis was further characterized by an increase in AD, but no change in RD.

**Figure 4:**
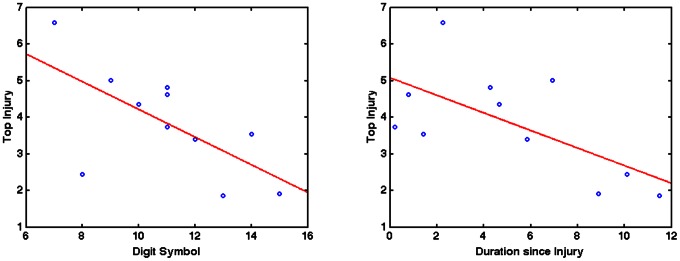
Correlating FA Severity with digit symbol and duration since injury. Correlation analysis of the Digit Symbol score and FA Severity (left) shows a negative relationship between the two measures indicating poorer performance on the Digit Symbol test associated with more abnormal FA. Duration since Injury and FA Severity (right) were also negatively correlated suggesting FA abnormalities become fewer as time progresses.

Similar to the Budde et al. study [17], we performed an AD versus RD analysis (**Figure 4**) that suggested two different patterns leading to increased FA. One is an increased AD with little or no change in RD, supporting the gliosis hypothesis. The other pattern, however, is a decrease in both AD and RD, which means that water diffusion is more hindered in these ROIs. To the best of our knowledge, a similar pattern of increased FA due to decreased diffusivities in GM has not been previously studied in the DTI literature. Candidate GM pathologies could be cytotoxic edema [33], neuroplasticities such as increased neurite density [34], the presence of plaques or other diffusion hindering material [35], or atrophy [16], [36], although atrophy is usually associated with increased diffusivities [16]. We further found a negative correlation between FA abnormalities and duration-since-injury, suggesting that FA abnormalities may be associated with a dynamic process, a finding in line with a recent study that observed changes in MD in subjects from two months to one year following TBI [37]. Therefore, the two different AD/RD patterns may also indicate two different time signatures of the gliosis process. Further studies are required to confirm the presence of gliosis in PPCS and its correlation with high AD in GM as well as the identification of the underlying mechanisms resulting in increased FA associated with reduced AD and RD.

There are also potential methodological sources for this observed FA increase in GM, and we performed two experiments to rule out some of them. First, we checked the reliability of FA in GM. We calculated the coefficient of variation (CV) of FA in both GM and WM, following the method of Farrell et al. (2007) [38]. We found that CV of FA was higher in GM than WM (t-test; p<0.01), but this variance was not significantly different between NC and PPCS in GM (t-test; p = 0.99) or WM (t-test; p = 0.94). Therefore, while the variability of FA was higher in GM than in WM, it was not biased towards either group. Second, we tested whether partial voluming effects in GM regions might have an impact on this result. Indeed, a higher WM partial volume in GM Freesurfer regions in PPCS compared to NC could explain our high FA observation. We tested for this possibility by eroding all GM regions via removal of all border voxels of each ROI. We then re-ran all subject-specific and group analyses. After GM erosion, our findings were unchanged and there remained a significant group differences between the PPCS and matched control groups when comparing the load (p = 0.023) and severity (p = 0.035) in GM regions. The observed differences using the original, non-eroded GM regions were p = 0.021 for load and p = 0.048 for severity.

With respect to WM, decreased FA reported in this study is consistent with findings reported in many mTBI studies [6]–[8]. Additionally, all of the WM abnormalities showed a significant increase in RD, which is consistent with DAI and demyelination processes [39]. However the many more GM abnormalities suggest that GM abnormalities may be more associated with longer-term symptoms and perhaps be a biomarker that predicts poorer outcome if present early in the course of mTBI.

One limitation of this study is the small sample size and its apparent heterogeneity, in particular its wide range in duration-from-injury. While it is difficult to draw conclusions regarding the relationship between symptoms, cognitive function and imaging data with this sample, this study does provide support for the robustness and sensitivity of the subject-specific analysis methodology. Although subject-specific tools have been proposed recently by other groups [32], [40], [41], our method is unique in that it allows for the investigation of DTI indices in both GM and WM ROIs. In addition, the use of an automated ROI-based analysis (via FreeSurfer) allows us to circumvent many of the problems associated with voxel-based analysis, in particular the errors stemming from imperfect non-linear registration of every subject into a common space.

In summary, using a subject-specific analysis on a PPCS cohort, we are able to consistently detect unique FA abnormalities in patients, but not in matched controls. Importantly, our findings suggest that TBI-related GM injury may be investigated not just with macroscopic measures of atrophy, but also with diffusion-related measures of tissue microstructure, shedding new light on the complex pathophysiology of PPCS. The finding of increased FA in GM calls for further investigation to verify the possibility of two different underlying pathologies characterized by changes in AD and RD and their link to clinical symptoms. Finally, the individualized analysis shows great promise for enhancing the clinical care of PPCS patients as it could ultimately play a role in the clinical diagnosis of structural brain injury that is not revealed using conventional MR.
